# Thermotolerance of *tax-2* Is Uncoupled From Life Span Extension and Influenced by Temperature During Development in *C. elegans*

**DOI:** 10.3389/fgene.2020.566948

**Published:** 2020-10-06

**Authors:** Ho-Yon Hwang, Laura Dankovich, Jiou Wang

**Affiliations:** ^1^Department of Biochemistry and Molecular Biology, Johns Hopkins University, Baltimore, MD, United States; ^2^Department of Neuroscience, Johns Hopkins University, Baltimore, MD, United States

**Keywords:** dauer survival, thermotaxis, cyclic nucleotide gated ion channel, insulin signaling pathway, heat shock factor

## Abstract

Thermotolerance of an organism is a complex trait that is influenced by a multitude of genetic and environmental factors. Many factors controlling thermotolerance in *Caenorhabditis elegans* are known to extend life. To understand the regulation of thermotolerance, we performed a genetic screen for mutants with better survival at warm temperature. Here we identified by dauer survival a *tax-2* mutation and several mutations disrupting an insulin signaling pathway including the *daf-2* gene. While the *tax-2* mutant has improved thermotolerance and long life span, the newly identified *daf-2* and other insulin signaling mutants, unlike the canonical *daf-2(e1370)*, do not show improved thermotolerance despite being long-lived. Examination of *tax-2* mutations and their mutant phenotypes suggest that the control of thermotolerance is not coupled with the control of life span or dauer survival. With genetic interaction studies, we concluded that *tax-2* has complex roles in life span and dauer survival and that *tax-2* is a negative regulator of thermotolerance independent of other known thermotolerance genes including those in the insulin signaling pathway. Moreover, cold growth temperature during development weakens the improved thermotolerance associated with *tax-2* and other thermotolerance-inducing mutations. Together, this study reveals previously unknown genetic and environmental factors controlling thermotolerance and their complex relationship with life span regulation.

## Introduction

The ability of a living organism to survive high temperature, or thermotolerance, could be related to molecular mechanisms of stress response and life span regulation. In the widely used genetic model *Caenorhabditis elegans*, thermotolerance genes also affect life span. The first thermotolerance mutation *age-1(hx546)*, which impairs phosphoinositide 3-kinase (PI3K) (Morris et al., 1996), was examined for thermotolerance because it confers resistance to oxidative stress and extends life span (Lithgow et al., 1994). Long life span was again a motivation behind the subsequent demonstration that mutations in *daf-2*, *daf-4*, and *daf-7* also confer improved thermotolerance (Lithgow et al., 1995). The *daf-2* insulin growth factor (IGF) receptor (Kimura et al., 1997) acts upstream of *age-1* in the insulin branch of dauer signaling pathway (Murphy and Hu, 2013), and many *daf-2* mutants are very long-lived (Van Voorhies, 1992; Kenyon et al., 1993). The *daf-7* transforming growth factor β (TGFβ) and *daf-4* type II receptor act in another genetic pathway referred to as group II dauer signaling pathway (Gumienny and Savage-Dunn, 2013), and mutations in *daf-4* and *daf-7* as well as other genes in the *daf-7* TGFβ pathway were later also found to confer extended life span (Shaw et al., 2007). Loss-of-function mutation in *daf-16* FOXO, which acts downstream of *daf-2* in the insulin branch dauer pathway, suppresses both life span extension and improved thermotolerance conferred by mutations in both the insulin branch and TGFβ dauer pathways (Hsu et al., 2003; Shaw et al., 2007). Another major focus in the study of *C. elegans* thermotolerance is the gene *hsf-1* heat shock transcription factor (Garigan et al., 2002). HSF-1 is notable because of its role in counteracting harmful effects of protein misfolding and aggregation resulting from stress (Akerfelt et al., 2010). Overexpression of full-length HSF-1 increases thermotolerance (Kourtis et al., 2012). This thermotolerance phenotype mirrors earlier findings in the role of *hsf-1* in life span, where overexpression of HSF-1 leads to life span extension and loss of *hsf-1* causes shortening of life span (Hsu et al., 2003). On the other hand, other reports suggest that the *daf-2* and *hsf-1* control of thermotolerance are not coupled to their control of life span (Wolff et al., 2006; McColl et al., 2010; Douglas et al., 2015), and the presence and the nature of shared underlying mechanism between these biological processes overall are unclear.

Improved thermotolerance in *C. elegans* also is associated with environmental factors, such as pretreatments using chemicals and heat. For example, heat pretreatments at 30°C for 3 to 24 h improve thermotolerance of *age-1* mutant and N2 wild type worms (Lithgow et al., 1995). Similar heat pretreatments also improve thermotolerance in overexpressors of *hsf-1* heat shock factor (McColl et al., 2010; Kourtis et al., 2012). Chemical treatments using hyperbaric oxygen (Cypser and Johnson, 2002), azide (Massie et al., 2003), blueberry polyphenols (Wilson et al., 2006), hydrogen sulfide (Miller and Roth, 2007), various antioxidants (Benedetti et al., 2008), trehalose (Honda et al., 2010), and L-arginine (Furuhashi et al., 2016) all improve thermotolerance. Such improvement in thermotolerance following heat and chemical pretreatments is often called induced thermotolerance, a byproduct of non-lethal stress generating anti-stress response. Induced thermotolerance is often thought distinct from the improved thermotolerance of mutants, which is considered intrinsic not requiring non-lethal stress. Whether there are shared cellular and molecular mechanisms between induced thermotolerance and intrinsic thermotolerance is unknown.

To gain insight into the regulation of thermotolerance, we performed a genetic screen for mutants that could reproduce at an inhospitably warm environment. Absent any reproducing mutants, we instead examined a mutant dauer survivor for improvement in thermotolerance. With this *tax-2* mutant showing signs of improved thermotolerance and long life span, we sought more mutant dauer survivors using the same screen and identified insulin signaling mutants including those defective in *daf-2*. Interestingly unlike the canonical *daf-2(e1370)* mutant, newly identified *daf-2* mutants do not show improved thermotolerance despite being long-lived. With examination of four different *tax-2* mutations, we found that the strengths of different mutant phenotypes do not match, which suggests that the control of underlying biological processes are not coupled. Genetic interaction studies with the companion gene *tax-4* suggest that stronger improvement in thermotolerance and weaker life span extension are likely null phenotypes, which indicates that *tax-2* has a complex role in life span extension. While examining the combined effect of *tax-2* and *daf-2* mutations in thermotolerance to determine whether the two genes act independently in controlling thermotolerance, we found that growth at 15°C eliminates much of improvements in thermotolerance normally seen following growth at standard 20°C. Furthermore, cold growth temperature of 15°C appears to have a broad weakening effect in the thermotolerance of most if not all known mutants with improved thermotolerance. Finally, temperature shift experiments were performed to further assess the role of growth temperature on thermotolerance associated with *tax-2* and other genes.

## Materials and Methods

### Strain Construction and Maintenance

*Caenorhabditis elegans* strains were cultured using standard methods (Brenner, 1974). Many isolates of same genotype were examined because of unexpected mutant phenotypes including quantitative differences in dauer phenotype. All available strains are listed in Supplementary Table 1. The genotype of strains was assessed by PCR using oligomer pairs designed to detect wild type or mutant alleles as listed in Supplementary Table 2. For genotype assessment, individual adult worms were lysed in 10 μl of 1× lysis buffer (40 mM KCl, 10 mM Tris pH 8.3, 2.5 mM MgCl_2_, 0.45% IGEPAL, 0.45% Tween 20, 60 μg/ml proteinase K) by incubation at 65°C for 1 h and 95°C for 15 min, and 2 μl of each lysate was used for PCR amplification with the cycling condition: 5′ 95°C, 35 cycles of (30′′ 95°C, 30′′ 60°C, 2′ 72°C), 5′ 72°C.

### Genetic Screen

Ethyl methanesulfonate (EMS) mutagenesis was performed as described (Brenner, 1974). Small number of synchronized F1 young adult hermaphrodites from single mutagenized worm was placed at 28°C. After 10 days at 28°C, nematode growth media (NGM) plates with reproducing survivors were sought. Total of 6,616 haploid genomes using N2 and 4,716 haploid genomes using *hsf-1(sy441)* were screened without success. Instead, we turned to a *tax-2(iw80)* dauer survivor derived from N2 at the end of these efforts. After seeing signs of improved thermotolerance in the *tax-2(iw80)* mutant, we screened 4,630 additional haploid genomes now looking for dauer survivors. Sixteen additional mutants were identified. Of the 16, four mutants (*iw87*, *iw94*, *iw95*, and *iw96*) were from the same EMS mutagenesis and have the same two mutations in *daf-2*. We suspect that *iw94* mutation was present in a subpopulation of worms prior to the EMS mutagenesis. Three mutants (*iw90*, *iw91*, and *iw92*) did not generate fertile adults reliably following 10 days at 28°C and were not pursued further. In all, 11 tractable mutants (*iw80* to *iw86*, *iw88*, *iw89*, *iw93*, and *iw94*) were kept.

### Mapping

The *iw80* mutation was mapped using long survival mutant phenotype at 28°C. Here, F3 mixed population derived from CB4856 mated with the *iw80* mutant were tested for long survival, and sister F3 plates of long surviving populations were used as the source of genomic DNA. With the other mutations, dauer survival mutant phenotype was used. Specifically, CB4856 males were mated with the mutant hermaphrodites, and three F1 L4 hermaphrodites were allowed to generate progeny at 20°C for 2 days. The resulting mix of F2 L1 larvae and embryo was subjected to 10 days of incubation at 28°C. Following 10 days at 28°C, the worms were moved to 20°C for recovery, and fertile adults were collected. Each recovered fertile adult assumed to be homozygous for the mutation of interest was used to produce more progeny, which served as the source of genomic DNA. PCR amplification was used to help detect DNA variants between N2 and CB4856 wild strains, mostly using “snip” SNP markers that could be distinguished using *Dra*I (Davis et al., 2005) or other restriction enzymes. The mapping results and additional details are provided in Supplementary Table 3.

### Outcrossing

Three approaches were used for outcrosses. For the first two *tax-2(iw80)* outcrosses, longer survival at 28°C starting with mixed population was used. For other outcrosses prior to candidate gene identification, survival as dauer larva at 28°C for 10 days and subsequent recovery to become fertile adult was used. Here akin to the method used for mapping, three potentially heterozygous L4 hermaphrodites were allowed to produce progeny at 20°C for 2 days prior to the start of 28°C incubation. After the candidate gene identification, detection of the molecular lesion of interest by PCR was used. All phenotypic characterizations reported here were performed using outcrossed strains except in the case of longer survival at 28°C with *iw80* mixed population.

### DNA Sequencing

With mutations mapped near *daf-2*, two largest exons of *daf-2* were sequenced following PCR amplification. With other mutations, whole-genome sequencing was performed. With the exception of a non-outcrossed *tax-2(iw80)* strain, all strains used for whole-genome sequencing were isolates that had undergone only a small number outcrosses and were not kept. Genomic DNA from non-outcrossed and outcrossed mutants was extracted using DNeasy Blood & Tissue Kit (Qiagen). Multiplexed libraries prepared using the genomic DNA were sequenced using Illumina HiSeq technology. Raw FASTQ files were aligned to build ws220 of the *C. elegans* genome using bowtie (Langmead, 2010), and mutations were identified using SAMtools (Li, 2011).

### Thermotolerance Assay

Mid-L4 hermaphrodites were preferentially used along with smaller number of early L4 larvae for all thermotolerance assays. Fifteen L4 hermaphrodites were transferred to NGM plates at room temperature. Room temperature was usually between 22 and 23°C, and results from transfers made at >25°C were discarded. After at least 30 min at room temperature following the completion of transfers, the plates were moved to 37°C. Stacking of plates was avoided at 37°C. The plates were taken out of 37°C after heat stress lasting from 1.5 to 4.5 h usually in 30-min intervals. The plates were left at room temperature overnight. Next day, worms subjected to the shortest duration of heat stress were examined first, and longest duration last. Otherwise, the order of examination for survival was determined by the order of transfer. Worms were examined using droplets of water, and completely immobile worms were scored as dead. Dead worms with vulval rupture, worms that died from climbing up the side of the plates, and missing worms were censored. We meticulously rotated the order of transfer to overcome a persistent artifact (Supplementary Figure 1). For each heat stress duration per genotype, at least ten examinations were made (Supplementary Table 4). For standard assays without temperature shift, all strains were cultured at 20°C for at least 3 days or at 15°C for at least 5 days prior to the heat treatment at 37°C.

For temperature shift experiments, plates with a small number of L4 larvae or adults in some cases for *hsf-1(sy441); uthIs225* mutants, which have high incidence of sterility, were initially placed at either 15 or 20°C. After spending a minimum of 2 days and a maximum of 13 days at the initial starting temperature, the plates were moved to warmer or cooler temperature for the temperature shift. For temperature shift 1 day prior to the lethal heat treatment, the time between temperature shift and the start of 37°C heat treatment ranged from 24 h 6 min to 26 h 34 min. For temperature shift 2 days prior to lethal heat treatment, the time between temperature shift and the start of 37°C heat treatment ranged from 46 h 37 min to 50 h 38 min with most at more than 49 h. For most experiments involving temperature shift 2 days prior to the lethal heat treatment, the mutants were at room temperature briefly the previous day because the same plates were also used for temperature shift 1 day prior to the heat treatment at 37°C.

### Dauer Survival Assay

An essential requirement of this assay is that worms do not run out of food. A small number of gravid adult hermaphrodites (usually eight) were allowed to lay eggs for a duration ranging from 40 to 300 min. All plates used for egg laying were freshly seeded with OP50 bacteria the previous day for ease in egg counting. After egg counting, most plates were moved to 20°C to allow eggs to hatch before the move to 28°C, and some plates with unhatched eggs were moved to 28°C the same day. After 10 days at 28°C, the plates were returned to 20°C. Starting after 3 days at 20°C, F1 fertile adults were counted and removed. We ensured that only F1 not F2 adults were counted by limiting counting of fertile adults to 2 days starting from the first day a fertile adult was spotted. Plates with L1 larvae but without an obvious F1 fertile adult were counted as having one F1 fertile adult. For our main analysis, we only used assays with 40 to 99 starting eggs and with plates that were moved to 28°C following 20 to 25 h of wait after the start of egg laying. Also for the main analysis, the data from different isolates with same genotype from different dates were combined, with a caveat that survival numbers could fluctuate quite a bit presumably because of varying conditions that could be difficult to control. Summary of dauer survival for different isolates and also by different dates are provided in Supplementary Table 5. The data from all individual assays are provided in Supplementary Table 6.

### Life Span Assay

For each assay, 15 L4 hermaphrodites were placed on a plate. Upon completion of worm transfer, the plates were placed at 28°C. Day 0 is the date of the transfer to 28°C as L4 larvae. After 3 to 5 days, the plates were monitored daily for most strains or every other day for some *daf-2* and *aap-1* mutants. All genotypes were tested using a minimum of five plates with at least three different starting dates (Supplementary Table 7), and the data from different isolates with same genotype were combined for the main analysis.

### Statistics

For analysis examining thermotolerance, comparisons using means and standard errors were supplemented with *p*-values obtained using generalized linear model assuming binomial distribution. For dauer survival rates, Student’s *t* test was used to obtain *p*-values. For examining life span, *p*-value of log rank test was obtained using R packages survival and survminer. For all three assays, data from many isolates of same genotype were combined. While no weighing was done to account for different data set sizes of different isolates, the difference in dataset sizes was small for thermotolerance and life span assays.

## Results

### A Genetic Screen Identifies a *tax-2* Mutation With Improved Thermotolerance

We started with a proposition that mutants that could reproduce at normally inhospitable temperature have improved thermotolerance and that a direct genetic screen of such mutant is feasible. To determine the upper limit of warm temperature for reproduction, we grew N2 worms at temperatures above 25°C, the standard warm culturing temperature. We also examined the *hsf-1(sy441)* loss-of-function mutant, which was thought to have decreased thermotolerance (Prahlad et al., 2008) and is largely devoid of heat shock protein (HSP) expression (Hajdu-Cronin et al., 2004; McColl et al., 2010). We found that N2 wild type worms could reproduce at temperatures up to 27°C. At 28°C, N2 worms lose the ability to reproduce whereas *hsf-1(sy441)* worms lose the ability to reproduce at 26.5°C. Therefore, we started a genetic screen looking for mutants that could reproduce at the restrictive temperature of 28°C starting with N2 and at 26.5°C starting with *hsf-1(sy441*) hoping to identify new thermotolerance genes.

**FIGURE 1:**
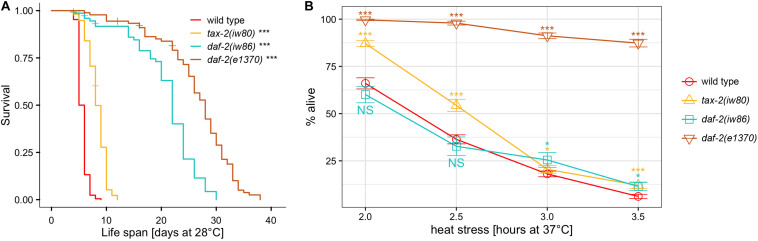
As compared to N2 wild type, the difference in the length of survival is different at 28°C and at 37°C with different mutants. Genotypes tested are N2 wild type, *tax-2(iw80)*, *daf-2(iw86)*, and *daf-2(e1370)*. **(A)** L4 hermaphrodites were placed at 28°C and were monitored for survival. **(B)** Mean percentage and standard error of survival of L4 larvae following heat stress at 37°C. Statistical comparison is against N2 wild type. ***p < 0.001, **p < 0.01, *p < 0.05. NS, not significant.

Following EMS mutagenesis, 6,616 haploid genomes starting with N2 strain and 4,716 haploid genomes starting with *hsf-1(sy441)* strain were screened without identifying any mutant capable of indefinite reproduction. However, starting with N2 but not with *hsf-1(sy441)*, we identified a mutant designated *iw80*, which persisted as dauer larvae even after 10 days at 28°C with abundant bacteria available as food. In the presence of abundant food, non-mutagenized N2 wild type worms never persist as dauer larvae for so long. After long survival at 28°C, *iw80* mutant dauer larvae following a move to 20°C could become fertile adults, which allows further characterization. We first tested non-outcrossed *iw80* mutants by placing embryos at 28°C and monitoring survival. While *iw80* mutants survive longer, many survive as dauer larvae for many days before becoming adults. To eliminate the complexity of dauer larvae, we monitored survival of outcrossed *iw80* mutants at 28°C starting instead with L4 larvae. With L4 larvae, the *iw80* mutants still show longer survival (Figure 1A, Table 1). However, the survival at 28°C lasts many days, which suggests that we are measuring life span, perhaps under stressful condition, rather than thermotolerance. Therefore, we also examined survival at 37°C, which leads to death within hours and thus should be a good test of thermotolerance. At 37°C, the *iw80* mutants show improved thermotolerance (Figure 1B). The *iw80* mutation was mapped (Supplementary Table 3) and sequenced, which identified a G423E (GGA to GAA) mutation in the gene *tax-2* ortholog of cyclic nucleotide-gated ion channel beta subunit (CNGB) (Coburn and Bargmann, 1996) and suggested that *tax-2* is a thermotolerance gene.

**TABLE 1:** Life span at 28°C.

Genotype	Median	0.95 LCL	0.95 UCL	Worms	Trials
*aap-1(iw89)*	16	16	16	75 (2)	5
*age-1(hx546)*	14	13	14	180	12
*daf-2(e1370)*	28	26	29	90 (9)	6
*daf-2(iw81)*	10	10	10	75	5
*daf-2(iw84)*	16	16	17	70 (1)	5
*daf-2(iw86)*	22	22	24	75 (5)	5
*daf-2(iw94)*	10	10	10	75	5
*tax-2(gk117937)*	7	7	7	90	6
*tax-2(gk117937); tax-4(p678)*	7	7	7	181 (1)	12
*tax-2(iw80)*	8	8	9	270 (2)	18
*tax-2(iw80); tax-4(p678)*	8	7	8	180 (2)	12
*tax-2(p671)*	7	7	7	165	11
*tax-2(p671); tax-4(p678)*	7	7	8	270 (2)	18
*tax-2(p691)*	8	8	8	194 (5)	13
*tax-4(p678)*	7	7	8	180 (1)	12
N2 wild type	5.5	5	6	212	15

*Shown are genotype, median life span in days, 0.95 lower and upper confidence limit, total number of worms examined (with number of censored), and number of trials.*

To obtain more mutants with similar characteristics as *tax-2(iw80)*, we continued the genetic screen with a modified aim of identifying mutants that could persist as dauer larvae for 10 days at 28°C. After screening 4,630 more haploid genomes, we identified ten tractable mutants *iw81* to *iw86*, *iw88*, *iw89*, *iw93*, and *iw94*. To identify the genes of interest in these new mutants, we performed genetic mapping (Supplementary Table 3) and DNA sequencing, and we determined that four of the mutants have mutations in the *daf-2* IGF receptor. Using Y55D5A.5a transcript predicted to generate a protein of 1,846 amino acids as the reference, the molecular changes are *iw81* L432F (CTT to TTT), *iw84* C490Y (TGT to TAT), *iw86* I1281F (ATT to TTT), and *iw94* A460T (GCG to ACG) and R1210H (CGC to CAC). Furthermore, a fifth mutant has a missense mutation *iw89* L55F (CTT to TTT) in the *aap-1* ortholog of phosphoinositide-3-kinase regulatory subunit 3 (PIK3R3), which partners with the PIK3 catalytic subunit encoded by *age-1* and acts downstream of *daf-2* in the insulin branch dauer pathway (Murphy and Hu, 2013). Notably, both *daf-2* and *age-1* are known thermotolerance genes (Lithgow et al., 1994, 1995). As was done earlier, we examined the life span of outcrossed mutants at 28°C starting with L4 larvae. All of these mutants show longer life span at 28°C (Figure 1A, Table 1, and Supplementary Figures 2A–C). On the other hand with heat stress at 37°C, to our surprise none of the mutants show improved thermotolerance (Figure 1B, Supplementary Figures 2D–F). In response, we examined the canonical *daf-2(e1370)* P1465S mutant. Here the *daf-2(e1370)* mutants show both long life span and improvement in thermotolerance (Figure 1). Interestingly using the same assays, the canonical *age-1(hx546)* P842S mutant does not show improvement in thermotolerance (Supplementary Figure 2G) but are long-lived (Supplementary Figure 2C). Given that *age-1(hx546)* adults previously were shown to have improved thermotolerance (Lithgow et al., 1994), it appears that the thermotolerance of L4 larvae and of adults could be different. Notably, only a small subset of insulin branch dauer pathway mutants shows strong improvement in thermotolerance as L4 larvae at 37°C despite all mutants showing longer life span at 28°C, which suggests a separation of the control of thermotolerance and the control of life span.

### *tax-2* Control of Thermotolerance Is Not Coupled With the Control of Life Span and Dauer Survival

**FIGURE 2:**
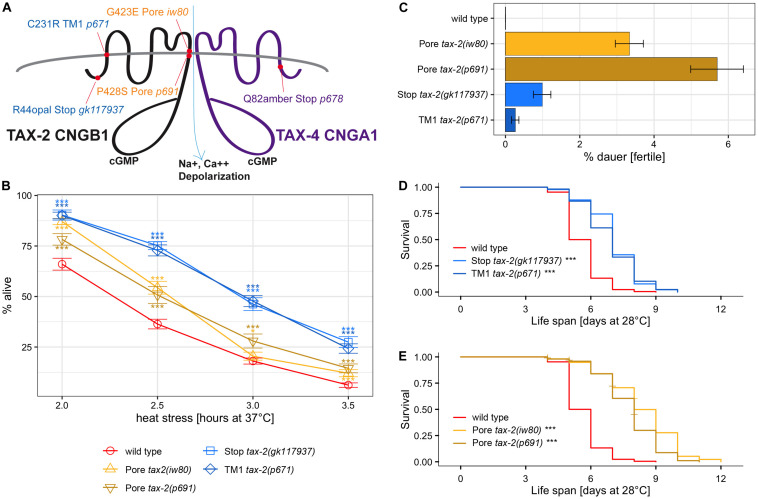
Absence of correlation between the strength of thermotolerance, dauer formation, and life span phenotypes among *tax-2* mutants. **(A)** Cartoon diagram of mutations of *tax-2* and *tax-4*. Mutations are *tax-2(gk117937)*, *tax-2(iw80)*, *tax-2(p671)*, *tax-2(p691)*, and *tax-4(p678)*. **(B)** Mean percentage and standard error of survival following heat stress at 37°C. Statistical comparison is against N2 wild type. **(C)** Mean percentage and standard error of dauer survival at 28°C. **(D–E)** Survival curve starting with L4 larvae at 28°C. Statistical comparison is against N2 wild type. Shown separately are **(D)** *tax-2(gk117937)* and *tax-2(p671)* with comparatively shorter life span than **(E)** *tax-2(iw80)* and *tax-2(p691)*. ***p < 0.001, **p < 0.01, *p < 0.05. NS, not significant.

The *tax-2* gene is associated with many aspects of *C. elegans* biology, including thermotaxis, chemotaxis, axon morphology, dauer development (Coburn and Bargmann, 1996; Coburn et al., 1998), life span (Apfeld and Kenyon, 1999), and detection of carbon dioxide (Bretscher et al., 2008) and light (Ramot et al., 2008). To confirm the effect of *tax-2(iw80)* on thermotolerance as well as life span and dauer survival observed here, three additional *tax-2* mutant alleles were examined. Among the four mutations, two mutations *tax-2(iw80)* G423E and *tax-2(p691)* P428S affect the TAX-2 channel pore (Figure 2A). While channel pore is critical to channel function, it is possible that altering the shape of channel pore merely changes the channel transport activity rather than completely disabling the channel. Another mutation *tax-2(p671)* C231R TM1 affects the first transmembrane domain. Disruption of the first transmembrane domain may cause mislocalization of the beta subunit and thereby prevent channel formation. The *tax-2(gk117937)* R44opal mutation, which has not been characterized previously, is likely a null allele with only 44 of 800 amino acids of the full-length protein retained. Careful examination of mutations and mutants could lead to better understanding of the relationship between the protein structure and function and the relationship between the gene and mutant phenotype.

To test for thermotolerance, we placed L4 larvae at 37°C for varying duration and determined survival the next day. Data from one to three different isolates of same genotype were combined for the comparison of different genotypes (Supplementary Tables 1,4). With *tax-2(gk117937)* R44opal and *tax-2(p671)* TM1 mutants, there is a robust improvement in survival (Figure 2B). Improved survival is less robust but significant with *tax-2(iw80)* and *tax-2(p691)* channel pore mutants. Channel pore mutations *iw80* and *p691* may be associated with weaker improvement in thermotolerance because some channel function is retained. The stronger improvement in thermotolerance in *gk117937* and *p671* mutants may be a result of strong or complete loss of the channel functions. These results confirm that *tax-2* is a bona fide thermotolerance gene.

Because our genetic screen depended on dauer survival, we also examined the strength of dauer survival. Here, synchronized population of L1 *tax-2* mutant larvae were placed at 28°C for 10 days, and the number of worms that became fertile adults upon return to 20°C was used to assess dauer survival rate. Dauer survival rate here reflects dauer formation as well as surviving as dauer larvae and recovering from dauer larvae. We found that dauer survival rates could be very different on different experiment dates, and dauer survival rates could differ for different isolates of same genotype from different outcrosses (Supplementary Tables 5,6). For instance, while the average dauer survival rate of *tax-2(p691)* mutant is higher than that of *tax-2(iw80)*, the dauer survival rate of *tax-2(iw80)* mutant is higher than that of *tax-2(p691)* mutant when both mutants were examined at the same time. All data combined, the dauer survival rate is higher with *tax-2(iw80)* and *tax-2(p691)* pore mutants than with *tax-2(gk117937)* R44opal and *tax-2(p671)* TM1 mutants (Figure 2C). Notably, the *tax-2* pore mutants with higher dauer survival rates have weaker improvement in thermotolerance. Thus the strength of dauer survival is the opposite of the strength of improvement in thermotolerance among the four *tax-2* mutations. The different strengths of the two mutant phenotypes suggest that the control of biological processes underlying the two phenotypes by *tax-2* is not coupled.

We also examined the *tax-2* mutants for their life span at 28°C starting as L4 larvae. Examining the four *tax-2* mutant alleles, all *tax-2* mutants show longer life span than wild type worms (Figures 2D,E, Table 1, and Supplementary Table 7). Notably the pore mutants *tax-2(iw80)* and *tax-2(p691)* have noticeably longer life span than *tax-2(gk117937)* R44opal and *tax-2(p671)* TM1 mutants. Recall that the *tax-2* pore mutants with longer life span have weaker improvement in thermotolerance. Thus as is the case with dauer survival, the strength of life span extension is the opposite of the strength of improvement in thermotolerance among the four *tax-2* mutations. Furthermore, these strengths of mutant phenotype suggest that the *tax-2* control of thermotolerance is uncoupled from the control of life span and the control of dauer survival.

### Loss of *tax-2* Improves Thermotolerance and Has Complicated Effects on Life Span and Dauer Survival

To confirm the *tax-2* model of uncoupled control of thermotolerance from other biological processes and to identify the null mutant phenotypes, we performed gene interaction studies using *tax-4* CNGA. CNG beta subunit encoded by *tax-2* is thought to form tetrameric ion channel complex together with CNG alpha subunits encoded by *tax-4*. Assuming that *tax-2* function always requires *tax-4*, null *tax-4* mutation should suppress *tax-2* gain-of-function mutation. Similarly, *tax-2* partial-loss-of-function *tax-4* null-mutation double mutants also should have the same phenotype as *tax-4* single null mutants. We used *tax-4(p678)* Q82amber mutation, which is likely a null mutation with only 82 of 733 amino acids of the full-length protein retained. Notably the *tax-4(p678)* mutation previously has been shown to confer improved thermotolerance (Cho et al., 2004). Knowing the null mutant phenotypes could inform how each mutation influences the relevant biological process.

**FIGURE 3:**
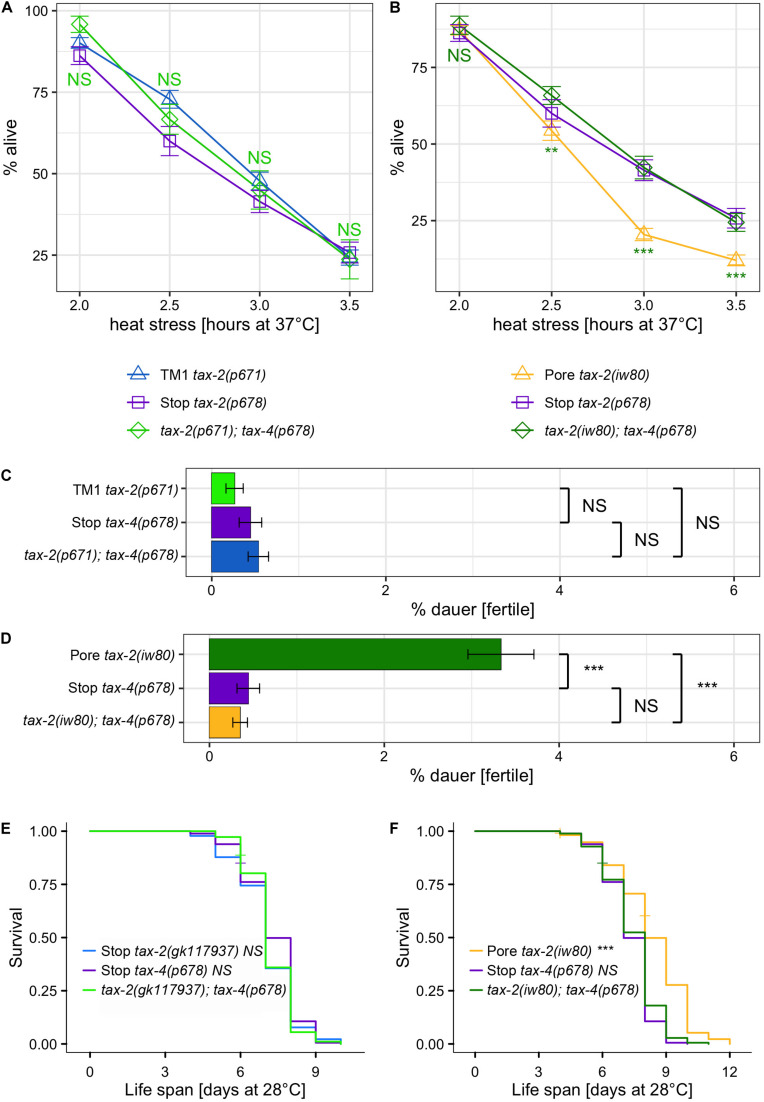
Examination of double mutants to determine *tax-2* null mutations. **(A,B)** Mean percentage and standard error of survival following heat stress at 37°C. Shown are **(A)** *tax-2(p671)*; *tax-4(p678)* double mutant and the single mutants with statistical comparison between the double mutant and *tax-2(p671)* single mutant and **(B)** *tax-2(iw80)*; *tax-4(p678)* double mutant and the single mutants with statistical comparison between the double mutant and *tax-2(iw80)* single mutant. **(C,D)** Mean percentage and standard error of dauer survival at 28°C. Shown are **(C)** *tax-2(p671)*; *tax-4(p678)* double mutant and the single mutants with statistical comparison against each other and **(D)** *tax-2(iw80)*, *tax-4(p678)* double mutant and the single mutants with statistical comparison against each other. **(E,F)** Survival curve starting with L4 larvae at 28°C. Shown are **(E)** *tax-2(gk117937)*; *tax-4(p678)* double mutant and the single mutants with statistical comparison against the double mutant and **(F)** *tax-2(iw80)*; *tax-4(p678)* double mutant and the single mutants with statistical comparison against the double mutant. ***p < 0.001, **p < 0.01, *p < 0.05. NS, not significant.

First, the strengths of thermotolerance were examined using single and double mutants of *tax-2* and *tax-4*. Both *tax-2(p671)* TM1 and *tax-4(p678)* Q82amber single mutants show improved thermotolerance indistinguishable from each other (Figure 3A). Furthermore, *tax-2(p671); tax-4(p678)* double mutants show similar strength of improved thermotolerance as the single mutants. The same levels of improvements in thermotolerance for all three genotypes are consistent with *p671* and *p678* being null mutations. Next, interactions with *tax-2(iw80)* pore mutation were examined. Here *tax-2(iw80)* pore single mutants show substantially worse improvement in thermotolerance than the *tax-4(p678)* single mutants (Figure 3B). Furthermore, *tax-2(iw80); tax-4(p678)* double mutants show similar improvement in thermotolerance as compared to *tax-4(p678)* single mutants. These results are consistent with *tax-2(iw80)* simply being a weak loss-of-function mutation. Other possibilities will be discussed later.

We also examined the effect of various *tax-2* mutations on dauer survival by comparing single and double mutants of *tax-2* and *tax-4*. All data combined, *tax-2(p671); tax-4(p678)* double mutants as well as both *tax-2(p671)* TM1 and *tax-4(p678)* Q82amber single mutants show similar dauer survival rates (Figure 3C, Supplementary Table 5), which is consistent with all these mutations being null mutations. The *tax-2(gk117937)* R44opal mutation also appears to be a null mutation according to a similar analysis (Supplementary Figure 3). On the other hand, *tax-2(iw80)* pore mutants show a stronger dauer survival than the *tax-4(p678)* single mutants (Figure 3D, Supplementary Table 5). Furthermore, *tax-2(iw80)* pore mutants show a stronger dauer dauer survival than the *tax-2(iw80); tax-4(p678)* double mutants. Since *tax-2(p678)* suppresses the high dauer survival rate associated with *tax-2(iw80)*, *tax-2(iw80)* could be a partial loss-of-function mutation. In addition to confirming the model of the *tax-2* control of thermotolerance and dauer survival being not coupled, these results suggest that the *tax-2* control of dauer survival is complicated.

Life span at 28°C was also examined using various single and double mutants of *tax-2* and *tax-4*. Here, *tax-4(p678)* single mutants have longer life span than wild type worms (Supplementary Figure 4A), and the life span of *tax-4(p678)* Q82amber single mutants is similar to those of *tax-2(p671)* TM1 and *tax-2(gk117937)* R44opal single mutants (Figure 3E, Supplementary Figures 4D–E). Furthermore, *tax-2(gk117937); tax-4(p678)* double mutants and *tax-2(p671); tax-4(p678)* double mutants both have life span of similar length as their single mutant counterparts (Figure 3E, Supplementary Figure 4B). On the other hand, *tax-2(iw80); tax-4(p678)* double mutants have shorter life span than *tax-2(iw80)* single mutants (Figure 3F). The life span of these double mutants all support the notion that *tax-2(p671)* TM1 and *tax-2(gk117937)* R44opal mutations are null mutations and that *tax-2(iw80)* pore mutation is not a null mutation. Furthermore, these results confirm that the *tax-2* control of thermotolerance is not coupled to the control of life span and suggest that the *tax-2* control of life span is complex.

### Loss of *tax-2* and *daf-2* Independently Improve Thermotolerance, Which Is Influenced Negatively by Cold Growth Temperature

To examine the relationship of *tax-2* with other thermotolerance genes, we compared double mutants with their single mutant counterparts. In cases where the mechanisms of thermotolerance are independent, the double mutant is expected to have a greater improvement in thermotolerance than the single mutants. In cases where the mechanism of thermotolerance associated with one gene is wholly a part of the mechanisms controlled by another gene, the double mutant is expected to have the same level of improvement in thermotolerance as the stronger of the single mutants. All other outcomes in the thermotolerance of the double mutants relative to the single mutants could indicate a complex genetic relationship. With such gene interaction studies, different thermotolerance genes could be sorted into common and distinct groups or genetic pathways.

**FIGURE 4:**
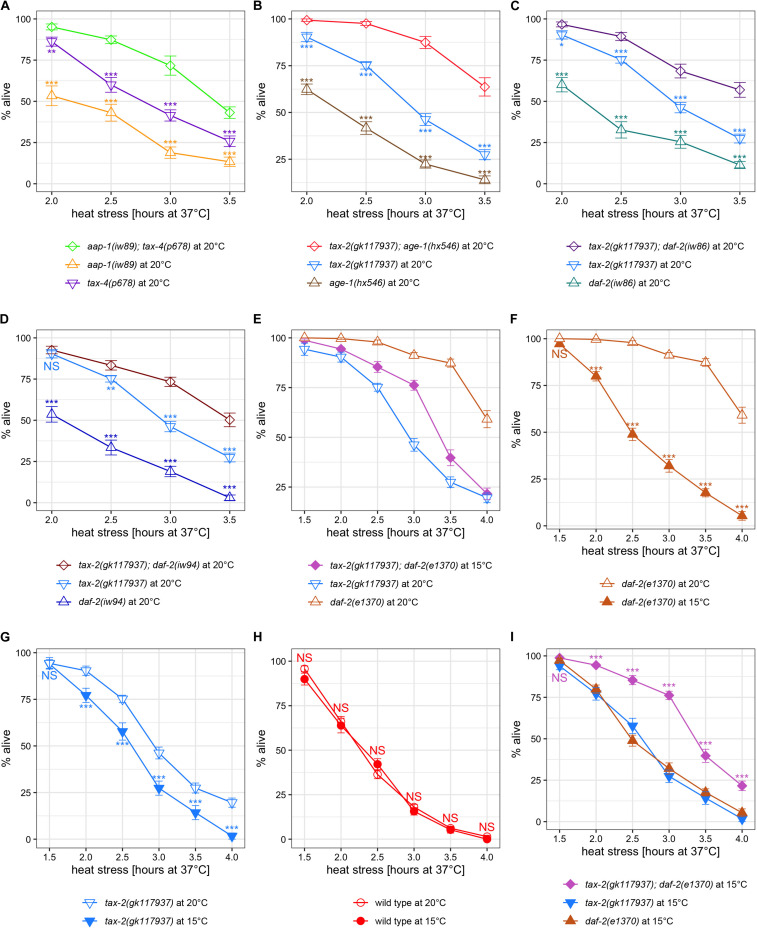
Examination of synergy in thermotolerance between insulin signaling mutations and *tax-2* and *tax-4* channel mutations led to a finding that growth temperature strongly influences thermotolerance. Mean percentage and standard error of survival following heat stress at 37°C. **(A)** *aap-1(iw89)*; *tax-4(p678)* double mutant is compared against the single mutants. **(B)** *tax-2(gk117937); age-1(hx546)* double mutant is compared against the single mutants. **(C)** *tax-2(gk117937); daf-2(iw86)* double mutant is compared against the single mutants. **(D)** *tax-2(gk117937); daf-2(iw94)* double mutant is compared against the single mutants. **(E)** *tax-2(gk117937); daf-2(e1370)* double mutant is compared against the single mutants. Here the double mutant is grown at 15°C whereas the single mutants are grown at 20°C. **(F)** *daf-2(e1370)* mutant grown at 20 and 15°C are compared. **(G)** *tax-2(gk117937)* mutant grown at 20 and 15°C are compared. **(H)** N2 wild type grown at 20 and 15°C are compared. **(I)** *tax-2(gk117937); daf-2(e1370)* double mutant is compared against the single mutants all grown at 15°C. ***p < 0.001, **p < 0.01, *p < 0.05. NS, not significant.

First, we examined the interaction between the *tax-2* and *tax-4* ion channel genes and the insulin branch dauer signaling pathway genes using weak mutations such as *aap-1(iw89)*, which by itself is not associated with improved thermotolerance in L4 larvae. Here *aap-1(iw89); tax-4(p678)* double mutants have stronger improvement in thermotolerance than the single mutant counterparts (Figure 4A). Similarly, *age-1(hx546); tax-2(gk117937)* double mutants, *tax-2(gk117937); daf-2(iw86)* double mutants, and *tax-2(gk117937); daf-2(iw94)* double mutants all have stronger improvement in thermotolerance than their single mutant counterparts (Figures 4B–D). Thus, weak mutations without detectable thermotolerance on their own could enhance thermotolerance associated with another mutation. Notably while *daf-2(iw86)* mutant is more than twice as long-lived as *daf-2(iw94)* single mutant at 28°C (Table 1, Supplementary Figure 2A), improvements in thermotolerance between *tax-2(gk117937); daf-2(iw86)* and *tax-2(gk117937); daf-2(iw94)* double mutants are indistinguishable (Supplementary Figure 6F), delineating thermotolerance from life span again. These enhancements suggest that *tax-2* and *tax-4* control of thermotolerance is independent from the insulin signaling pathway genes.

A different initial observation was made using *tax-2* double mutants with stronger *daf-2(e1370)* mutation. Notably, *tax-2(gk117937); daf-2(e1370)* double mutants become dauer larvae irreversibly at 20°C and thus need to be grown at 15°C to measure the thermotolerance of L4 larvae. Therefore, we initially compared thermotolerance of double mutants grown at 15°C with single mutants grown at 20°C, which is our standard growth temperature. Here, the *tax-2; daf-2* double mutants have an intermediate level of survival as compared to the single mutants (Figure 4E), which is difficult to explain.

To eliminate the possibility of growth temperature affecting thermotolerance, we repeated thermotolerance assays using single mutants grown at 15°C. Surprisingly, *daf-2(e1370)* mutants grown at 15°C show substantially worse survival than the same mutants grown at 20°C (Figure 4F). The *tax-2(gk117937)* mutants also show worse survival when grown at 15°C rather than at 20°C (Figure 4G). On the other hand, N2 wild type worms grown at 15°C show similar level of survival as N2 worms grown at 20°C (Figure 4H). Thus, cold growth temperature leads to dramatic reduction in the improved thermotolerance of *daf-2* and *tax-2* mutants but does not affect the thermotolerance of wild type worms.

Comparing only the strains grown at 15°C, both *daf-2(e1370)* single mutants and *tax-2(gk117937)* single mutants both show mild improvement in thermotolerance as compared to N2 wild type (Supplementary Figure 5). Importantly using the same cold growth temperature, *tax-2(gk117937); daf-2(e1370)* double mutants show substantially improved thermotolerance as compared to the single mutants (Figure 4I), which indicates a synergistic effect. Such synergistic effect is consistent with the control of thermotolerance associated with *tax-2* and *daf-2* being independent.

### Improvements in Thermotolerance by Many Genetic Factors Are Enhanced by Loss of *tax-2* and Are Negatively Influenced by Cold Growth Temperature

To understand the role of other known genetic factors of thermotolerance relative to *tax-2*, we performed more gene interaction studies. The other mutations associated with improved thermotolerance and used are *daf-7(e1372)* TGFβ, *spe-26(hc138)* (Lithgow et al., 1995), *ndg-4* (Brejning et al., 2014), and *uthIs225* [*hsf-1(CT)*]. Here, *uthIs225* [*hsf-1(CT)*] is an overexpressor of HSF-1 heat shock transcription factor with *sy441* mutation and thus without the *C*-terminal domain, which is necessary for activating downstream heat shock factors but dispensable in improving thermotolerance (Baird et al., 2014). In addition, we examined these mutants following growth at 15°C to see if the cold growth temperature effect is generalizable.

**FIGURE 5:**
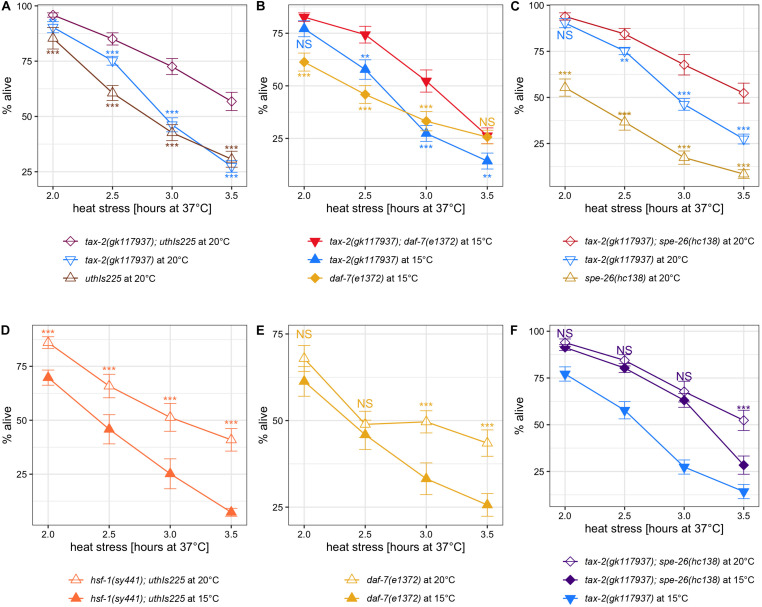
Other known thermotolerance mutations synergize with *tax-2* mutation and are influenced by growth temperature. Mean percentage and standard error of survival following heat stress at 37°C. **(A)** *tax-2(gk117937); uthIs225* [*hsf-1(CT)*] double mutant is compared against the single mutants. **(B)** *tax-2(gk117937); daf-7(e1372)* double mutant is compared against the single mutants. **(C)** *tax-2(gk117937); spe-26(hc138)* double mutant is compared against the single mutants. **(D)** *hsf-1(sy441); uthIs225* [*hsf-1(CT)*] grown at 20 and 15°C are compared. **(E)** *daf-7(e1372)* grown at 20 and 15°C are compared. **(F)** *tax-2(gk117937); spe-26(hc138)* grown at 20 and 15°C are compared against each other with *tax-2(gk117937)* grown at 15°C also shown. ***p < 0.001, **p < 0.01, *p < 0.05. NS, not significant.

Examining the interactions, *tax-2(gk117937); uthIs225* [*hsf-1(CT)*] double mutants show stronger improvement in thermotolerance than both *tax-2(gk117937)* single and *uthIs225 [hsf-1(CT)]* single mutants (Figure 5A). Stronger improvement in thermotolerance is evident also in the double mutants of *tax-2(gk117937); daf-7(e1372)* (Figure 5B) and of *tax-2(gk117937); spe-26(hc138)* (Figure 5C). While stronger improvement is less convincing in *tax-2(gk117937); ndg-4(gk823291)* double mutant (Supplementary Figure 6A), *ndg-4(gk823291)* single mutant shows at most a mild improvement in thermotolerance as L4 larvae (Supplementary Figure 6B). The *ndg-4(gk823291)* Q233amber mutant is good null candidate on paper, and we suspect that *ndg-4* has little influence in the thermotolerance of L4 larvae. These synergistic effects suggest that loss of *tax-2* influences thermotolerance independently of uthIs225 *[hsf-1(CT)]* or loss of *daf-7* and *spe-26*.

Examining the effect of cold growth temperature, *hsf-1(sy441); uthIs225 [hsf-1(CT)]* mutant shows a worse thermotolerance when grown at 15°C as opposed to 20°C (Figure 5D). Worse thermotolerance when grown at 15°C was evident also with *daf-7(e1372)* TGFβ mutant with longer heat stresses (Figure 5E). We note that *daf-7(e1372)* mutant shows weak to zero improvement in thermotolerance with shorter heat stresses (Supplementary Figures 5D, 6D). Because *spe-26(hc138)* single mutant does not show appreciable improvement in thermotolerance, we instead examined *tax-2(gk117937); spe-26(hc138)* double mutant. Here cold growth temperature appears to have an effect only with long heat stress in *tax-2(gk117937); spe-26(hc138)* mutant (Figure 5F). In summary, cold growth temperature has a broad effect on the thermotolerance associated with *hsf-1(sy441); uthIs225 [hsf-1(CT)]*, *daf-7* TGFβ, and possibly *spe-26*.

### Temperature Influences Thermotolerance Throughout Development, and Different Mutants Respond Differently to the Changes in Temperature

**FIGURE 6:**
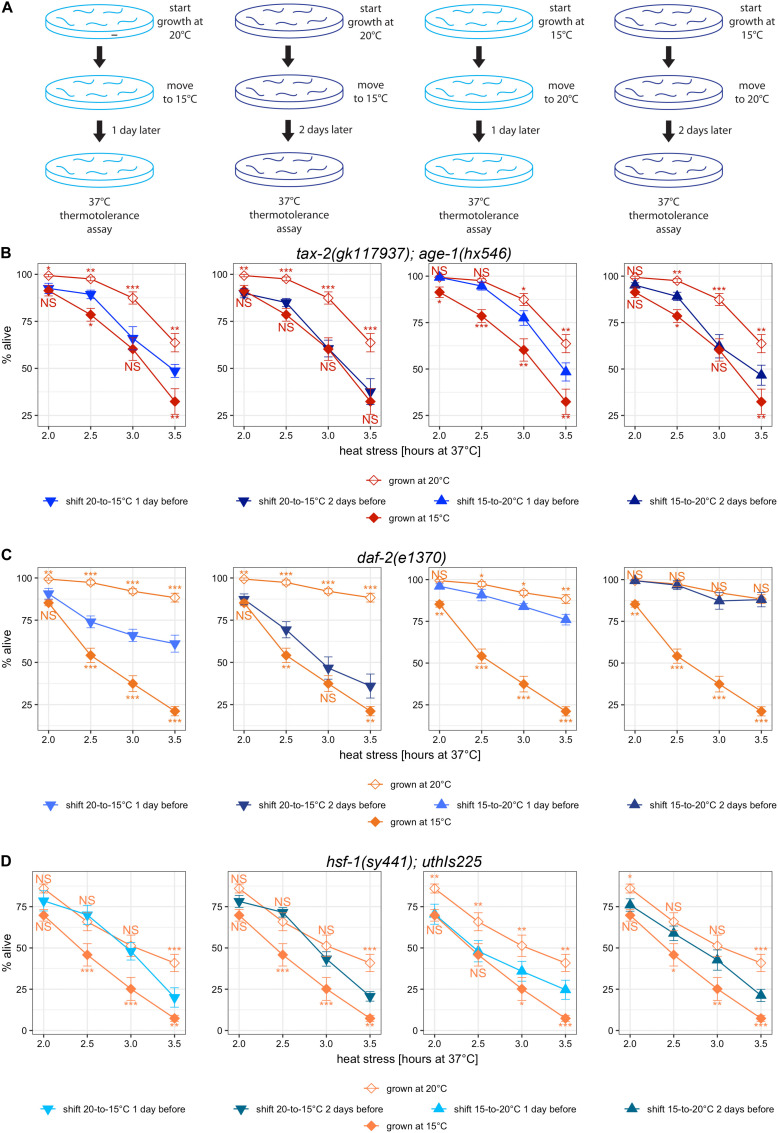
Temperature-shifts during development lead to different changes in thermotolerance with different mutants. **(A)** Cartoon diagram of the experimental procedure for temperature shifts. Worms initially cultured at 20°C were down-shifted to 15°C 1 or 2 days prior to the heat stress at 37°C. Worms initially cultured at 15°C were up-shifted to 20°C 1 or 2 days prior to the heat stress at 37°C **(B–D)** Mean percentage and standard error of survival following heat stress at 37°C. Shown are temperature-shifted worms compared against worms grown at same temperature throughout development. Mutant genotypes are **(A)** *tax-2(gk117937); age-1(hx546)* double mutant, **(B)** *daf-2(e1370)* mutant, and **(C)** *hsf-1(sy441); uthIs225* [*hsf-1(CT)*]. Statistical comparison is with worms that were grown at same temperature continuously against worms that were subjected to temperature shift. ***p < 0.001, **p < 0.01, *p < 0.05. NS, not significant.

To determine whether a specific period in development is critical for the cold growth temperature effect on thermotolerance, we performed a series of temperature shift experiments. *A priori*, the critical period could be a specific developmental stage or many different developmental stages. Alternatively, only the recent past or the temperature of acclimatization could be important. The general experimental outline is to grow mutants starting at either 15 or 20°C and then shift the temperature either a day or two prior to the lethal heat treatment using L4 larvae (Figure 6A). One day prior to the start of lethal heat stress, we estimate that a wild type worm at 20°C on average is a larva at the transition between L1 and L2 larval stages. Two days prior, a typical wild type worm at 20°C may be an embryo at gastrulation. Many mutants are slower growing, and cooler growth temperature slow development. Thus all things considered, a conservative estimate of the range of developmental stages at the point of temperature shift is between L1 and L2 larval stages one day prior to heat stress and between early embryonic and late embryonic stages two days prior to heat stress. In choosing strains, we wanted to be able to distinguish an intermediate improvement in thermotolerance from the thermotolerance with continuous growth at single temperature, which would be easier with strains with big growth temperature effect. Thus, we examined *tax-2(gk117937); age-1(hx546)* double mutant along with *daf-2(e1370)* single mutant and *hsf-1(sy441); uthIs225* [*hsf-1(CT)*] mutant - [*hsf-1(CT)*]. Differences in the response to the timing of temperature shift could be indicative of the differences in the mechanisms underlying the thermotolerance associated with each gene.

First, we examined *tax-2(gk117937); age-1(hx546)* mutants that started growth at 20°C and were subsequently moved to 15°C. Compared to the mutants grown continuously at 15°C, the mutants subjected to the downward temperature shift one day prior to heat stress have stronger improvement in thermotolerance (Figure 6B, leftmost panel). With a temperature shift to 15°C 2 days prior to lethal heat treatment instead, there is little difference in thermotolerance as compared to the mutants grown continuously at 15°C (Figure 6B, second panel). This suggests that the temperature during development spanning embryonic to late larval development contributes to improving thermotolerance.

Next, we examined *tax-2(gk117937); age-1(hx546)* mutants that started growth at 15°C and were subsequently moved to 20°C. Compared to the mutants grown continuously at 15°C, the mutants subjected to the upward temperature shift one day prior to heat stress show a much stronger improvement in thermotolerance (Figure 6B, third panel). However, with the upward temperature shift two days prior to lethal heat treatment instead, there is no further improvement in thermotolerance (Figure 6B, fourth panel). These upshift experiments suggest that cold growth temperature during embryonic and early larval development of *tax-2(gk117937); age-1(hx546)* mutants prevents acquiring thermotolerance at full-strength.

The effect of temperature shift was examined also using *daf-2(e1370)* mutants. With downshifts in temperature, thermotolerance after 2 days at 15°C is still stronger than the thermotolerance of those grown continuously at 15°C (Figure 6C, first two panels). With upshifts in temperature, thermotolerance after 2 days at 20°C is indistinguishable from the thermotolerance of those grown continuously at 20°C, and substantial gain is already visible with only 1 day at 20°C (Figure 6C, last two panels). Thus the effect of warm temperature during early development appears to last for considerable amount of time, and warm temperature during larval development may be sufficient for full-strength improvement in thermotolerance. Importantly, the effect of cold growth temperature during the development in in *daf-2(e1370)* mutants is different as compared to the effect in *tax-2(gk117937); age-1(hx546)* mutants, suggestive of some underlying differences between the thermotolerance of *tax-2* and *daf-2*.

Finally *hsf-1(sy441); uthIs225* [*hsf-1(CT)*] mutants were examined following temperature shifts. With downshifts in temperature, there is significant loss of improved thermotolerance arguably only with longer heat treatment, and the timing of temperature shift between 1 day and 2 days did not make an appreciable difference (Figure 6D, first two panels). With upshifts in temperature, there is a gradual increase in the improvement of thermotolerance without reaching the level of the mutants grown entirely at warmer temperature (Figure 6D, last two panels). In summary, improvements in thermotolerance gained during warmer temperature persist for a very long time in *hsf-1(sy441)*; *uthIs225* [*hsf-1(CT)*] mutants, and the improvements are gained very slowly throughout development. Importantly, the effect of temperature shift on *hsf-1(sy441)*; *uthIs225* [*hsf-1(CT)*] mutants is different from the effects on both *tax-2* and *daf-2* mutants.

## Discussion

This manuscript addresses the role of *tax-2* in dauer development and life span in addition to a previously undescribed role in thermotolerance using methods that are somewhat different as compared to methods used by others. The changes in methods could be complicating and beneficial. For example, our dauer survival assay measures multiple aspects of dauer biology, including dauer formation, maintenance, and survival as well as recovery to fertility. On the positive side, dauer survival over long time mitigates the complications stemming from the transient nature of dauer formation at 28°C, and big fertile adults are easier to count than small thin dauer larvae. With thermotolerance assay at 37°C, we started using L4 larvae in part because the shape of vulvae allows precise age determination as compared to the age of adults. We continued using L4 larvae also because of shorter length of heat stress needed to kill wild type and mutant worms. We note that there are differences in the thermotolerance of L4 larvae and adults, with *age-1(hx546)* being a clear example. Also while *daf-2(e1370)* L4 larvae grown at 15°C here show only a modest improvement in thermotolerance, a previous report showed that adult *daf-2(e1370)* grown at 15°C have strong thermotolerance as compared to N2 wild type (Gems et al., 1998). On the positive side with the life span assay at 28°C, reproduction is essentially eliminated at 28°C, which means that fewer worm transfers are needed. As for the lingering possibility of a thermotolerance component in life span assay at 28°C, we need a longer discussion.

To examine the possibility of a thermotolerance component in the life span assay at 28°C, we examined older reports of life span assays at different temperatures. Much data exists for widely studied N2 wild type and *daf-2(e1370)*. With *daf-2(e1370)*, the earliest reported mean or median life spans are 22.5 days at 25°C (Kenyon et al., 1993), 20.4 days at 22.5°C (Gems et al., 1998), 42 days at 20°C (Kenyon et al., 1993), and 33.8 days at 15°C (Gems et al., 1998). Subsequent reports as a whole also indicate longest life span at 20°C with mildly shorter life span at 15°C and even shorter life span at temperatures ranging from 22.5 to 25.5°C (Supplementary Tables 8,9). In comparison, median life span of 28 days at 28°C here is in fact longer than most reported life spans at temperatures ranging from 22.5 to 25.5°C. With N2 wild type, the earliest reported mean or median life spans are 17 days at 25°C (Kenyon et al., 1993), 16.3 days at 22.5°C (Gems et al., 1998), 20 days at 20°C (Kenyon et al., 1993), and 24 days at 15°C (Gems et al., 1998). Combined with later reports, the trend is a gradual lengthening of life span from 25 to 15°C (Supplementary Tables 8,9). In comparison, median life span of 5.5 days at 28°C here is considerably shorter. With *tax-2* and *tax-4*, the data is sporadic with only *tax-2(p671)* examined at many temperatures. The reported mean or estimated median life spans of *tax-2(p671)* mutants are 36 days at 15°C (Artan et al., 2016), 20 days at 20°C (Apfeld and Kenyon, 1999), 13 days at 25°C (Lee and Kenyon, 2009), and 7 days at 28°C here. Thus, there is a pattern of steady steep decline in the length of life span from cooler temperature to warmer temperature. Curiously, *tax-2* and *tax-4* mutants also have shorter life span than N2 wild type at 25°C (Lee and Kenyon, 2009), which is difficult to explain. In summary, while there is an atypical difference in life span between 25 and 28°C with N2 wild type, such big difference does not exist with *daf-2(e1370)* or with *tax-2(p671)*. Therefore, a simple thermotolerance component present at 28°C and absent at cooler temperatures seems incompatible with the contrasting trends in life span with different genotypes and temperatures.

Focusing on the nature of *tax-2* mutations and their effect, the best null allele candidates are *tax-2(gk117937)* and *tax-2(p671)*. As for *tax-2(iw80)* and *tax-2(p691)* pore mutations, we think that weak loss-of-function and gain-of-function mutations are both possible with weak loss-of-function being more likely. Exploring the gain-of-function possibility, a gain-of-function phenotype may be caused by increased activity (hypermorph), suppressed activity (antimorph), or new activity (neomorph). The hypermorph option is unlikely to be true because *tax-2(p691)* was shown to lack channel activity in some neurons (Ward et al., 2008). The antimorph option does not make sense because stronger mutant phenotypes of both life span and dauer formation as measured by survival are eliminated by the loss of *tax-4* heteromeric channel partner. On the other hand, we cannot rule out the neomorph option. Assuming that *tax-2(iw80)* and *tax-2(p691)* have neomorph function, our results suggest that *tax-4* is an important partner in this new function, probably as a channel. For example, *tax-2(iw80)* and *tax-2(p691)* mutation could lead to constitutive ion leakage.

Reported measurements of ion channel activities *in vivo* using *tax-2* and *tax-4* mutants are particularly relevant here. Ion channel activities in response to heat are eliminated in the major thermosensory neuron AFD in *tax-2(p671)* and *tax-4(p678)* mutants (Ramot et al., 2008), which is consistent with these mutations being null mutations. Ion channel activities in response to light in the major photoreceptor neuron ASJ were also eliminated in *tax-2(p671)* mutants (Ward et al., 2008). Interestingly, *tax-2(p691)* channel pore mutants also lack the same ion channel activities, which indicates that *tax-2(p691)* in ASJ behaves like a loss-of-function mutation and arguably a null mutation. Thus regardless whether a specific *tax-2(p691)* mutant phenotype is caused by loss-of-function or gain-of-function, it is almost certain that *tax-2(p691)* is at least a partial loss-of-function mutation. One possible model is that the ion channel activity of the mutant TAX-2 protein encoded by *tax-2(p691)* is eliminated in some neurons, such as ASJ, but not in other neurons, possibly including AFD.

Study of the neuronal circuitry of *C. elegans* by laser ablation has been informative for both life span and dauer formation (Bargmann, 2006). According to ablation studies, ASJ promotes dauer formation in the presence of pheromone whereas ASI and ASG prevent dauer formation (Bargmann and Horvitz, 1991; Schackwitz et al., 1996). Furthermore, ablation of ASI and ASG leads to longer life span whereas ablation of ASJ leads to mild shortening of life span (Alcedo and Kenyon, 2004). Both *tax-2* and *tax-4* are expressed in all three neurons ASJ, ASI, and ASG (Coburn et al., 1998; Birnby et al., 2000). These expression patterns suggest that these two genes could have dauer formation and suppression function as well as life span extension and shortening function. Having such opposing functions in these biological processes could explain the different strengths of dauer and life span mutant phenotypes in various *tax-2* mutants. For example, mutant TAX-2 protein with *iw80* mutation might be active in dauer-promoting neurons but inactive in dauer-suppressing neurons whereas mutant TAX-2 protein from *gk117937* null mutation is inactive in all neurons. At least two other groups also have proposed and discussed the possibility of *tax-2* and *tax-4* having opposing functions in dauer larva formation invoking different neuronal sites of action (Coburn et al., 1998; Ailion and Thomas, 2000). To reiterate, stronger dauer formation and longer life span of *iw80* and *p691* pore mutants as compared to those of null mutants may be explained by selective retention of channel function in neurons.

While the neuronal sites of action seem important in determining dauer formation and life span with *tax-2*, so far there is no evidence suggesting a similar model for thermotolerance. It is possible that *tax-2* could influence thermotolerance by the channel function activating downstream effectors. Alternatively given that a major function of *tax-2* is sensing temperature change, a thermotolerance model without invoking downstream effectors also should be considered. Absent other supporting evidences, mutant phenotypes are the best guide. Here null mutants of CNGB *tax-2* and CNGA *tax-4* have strong thermotolerance whereas *tax-2* pore mutants have weak thermotolerance, which may mean that these genes have simple roles in thermotolerance. On the other hand, a candidate null mutant of another CNGA *cng-3*, which seems likely to form a heteromeric ion channel, was shown to have worse thermotolerance than N2 wild type (Cho et al., 2004). The same report also showed that *tax-4* CNGA null mutation suppresses the worse thermotolerance phenotype associated with the *cng-3* CNGA mutation. While the interaction between *tax-4* and *cng-3* needs more clarification, CNG ion channels as it stands appears to have both positive and negative influence on thermotolerance.

Despite many connections between thermotolerance and life span, our examination of *tax-2* and *tax-4* mutations and their interactions show that these genes influence the two biological measures differently. Delineation of the roles in thermotolerance and life span could be seen also with mutants defective in *daf-2* and *aap-1* insulin pathway genes. The *daf-2* and *aap-1* insulin pathway mutants with substantial life span extension but without improvement in thermotolerance in this manuscript are reminiscent of *daf-2(e1370)* mutants with RNAi inactivation of *smk-1* protein phosphatase 4 regulatory subunit (Wolff et al., 2006) and *ctsa-3.2* cathepsin A (McColl et al., 2010). Similar idea separating life span and thermotolerance has been proposed also in the study of heat shock transcription factor HSF-1, which is thought to act in distinct neural circuits to regulate aging, which also requires *daf-16* FOXO in intestines, separately from thermotolerance (Douglas et al., 2015). Such different outcomes in life span and thermotolerance suggest that major differences exist in the mechanisms underlying these biological processes and that these biological processes are not interdependent.

Addressing the effect of growth temperature in general, we have shown that cold growth temperature weakens the improved thermotolerance of many *C. elegans* mutants. This weakening may be blurring the line between intrinsic thermotolerance and induced thermotolerance. Perhaps this weakening is closely related to the induced improvement in thermotolerance by heat pretreatment (Lithgow et al., 1995; McColl et al., 2010; Kourtis et al., 2012). Perhaps all these observations stem from a continuum of temperature effect. After all, 15°C could be the normal growth temperature for worms in the wild environment, and 20°C could be uncomfortably warm and stressful for many mutants. Using a similar reasoning, the distinction between intrinsic thermotolerance of mutants and induced thermotolerance by chemical treatments (Cypser and Johnson, 2002; Massie et al., 2003; Wilson et al., 2006; Miller and Roth, 2007; Benedetti et al., 2008; Honda et al., 2010; Furuhashi et al., 2016) may be not especially meaningful.

We also highlight more notable outcomes from the temperature-shift experiments. First we find it interesting that downshifted *hsf-1(sy441)*; *uthIs225* [*hsf-1(CT)*] mutants retain much of the improved thermotolerance acquired during early development at warmer temperature whereas *daf-2(e1370)* mutants and *tax-2(gk117937); age-1(hx546)* mutants lose improved thermotolerance more rapidly. This may be indicative of longer half-life of downstream thermotolerance factors of *hsf-1(sy441)*; *uthIs225* [*hsf-1(CT)*] mutant as compared to the downstream factors of the loss-of-function mutants. Next, while these presumed downstream thermotolerance factors slowly accumulate throughout most if not all phases of development, the rate of accumulation seems different with different genes. We find it striking that *daf-2(e1370)* mutant with the strongest improvement in thermotolerance requires the least amount of time to gain full improvement in thermotolerance during later development. Combined with gene interaction analysis, these distinct responses to the direction and timing of temperature shift all indicate that the thermotolerance mechanism underlying *tax-2* is likely at least partly independent of the mechanism controlled by other genes.

In all, *tax-2* is a thermotolerance gene with complex roles in life span and dauer survival, with temperature playing an important role in controlling all of these biological processes. We showed that loss of *tax-2* in *C. elegans* leads to improved thermotolerance, which suggests that *tax-2* normally has a negative influence on thermotolerance. Mutant phenotype characterizations indicate that the control of thermotolerance by *tax-2* is not coupled to the control of life span and the control of dauer survival, with *tax-2* likely influencing the latter two processes in both positive and negative manner. Gene interaction studies suggest that the *tax-2* controls thermotolerance independently of other known thermotolerance genes, including *daf-2* and *hsf-1*. In addition, temperature during development strongly influences the thermotolerance of *tax-2* and many other mutants. Temperature shift experiments suggest that different downstream thermotolerance effectors associated with *tax-2* mutation and mutations in other genes likely exist, each with different timing of expression control and with different rates of decay. We think that much remains to be examined and understood about the nature of thermotolerance and that dauer mutants could serve as a rich source of mutants affecting thermotolerance.

## Data Availability Statement

The DNA sequencing data is available at NCBI, accession: PRJNA659288.

## Author Contributions

H-YH and JW designed research and wrote the manuscript. H-YH analyzed data. H-YH and LD performed research. All authors contributed to the article and approved the submitted version.

## Conflict of Interest

The authors declare that the research was conducted in the absence of any commercial or financial relationships that could be construed as a potential conflict of interest.

## Abbreviations

AGE: ageing alteration
CNGA: cyclic nucleotide-gated ion channel alpha subunit
CNGB: cyclic nucleotide-gated ion channel beta subunit
DAF: abnormal dauer formation
EMS: ethyl methanesulfonate
HSF: heat shock factor
HSP: heat shock protein
IGF: insulin growth factor
NDG: Nordihydroguaiaretic acid resistant
NGM: nematode growth media
PCR: polymerase chain reaction
PI3K: phosphoinositide 3-kinase
PIK3R3: phosphoinositide-3-kinase regulatory subunit 3
RNAi: RNA mediated interference
SPE: defective spermatogenesis
TAX: abnormal chemotaxis
TGF β: transforming growth factor beta
TM1: transmembrane domain 1.
