# Supplementary figures and images for: Thermotolerance of tax-2 Is Uncoupled From Life Span Extension and Influenced by Temperature During Development in C. elegans
Source: Front Genet. 2020 Oct 6;11:566948. doi: 10.3389/fgene.2020.566948 (PMC7573314; doi:10.3389/fgene.2020.566948)

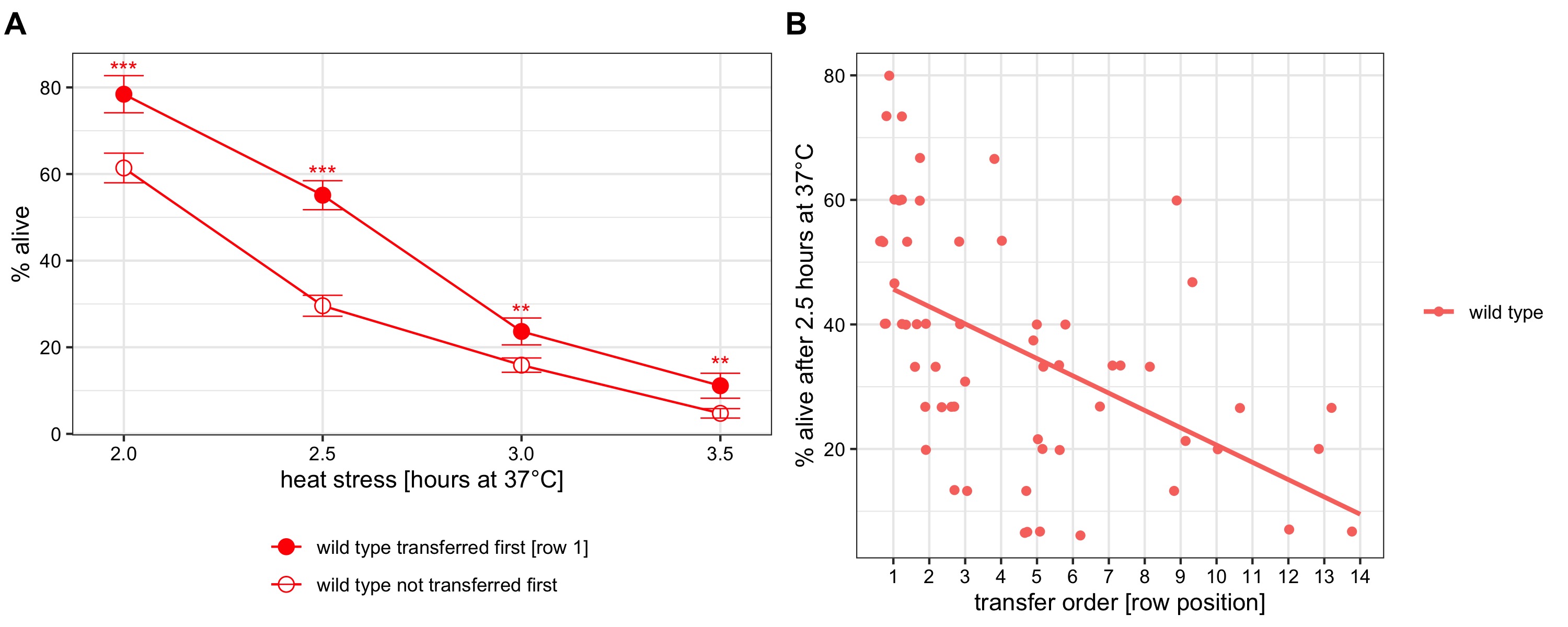

Supplement: Supplementary file 1 [file Image_1.jpg]

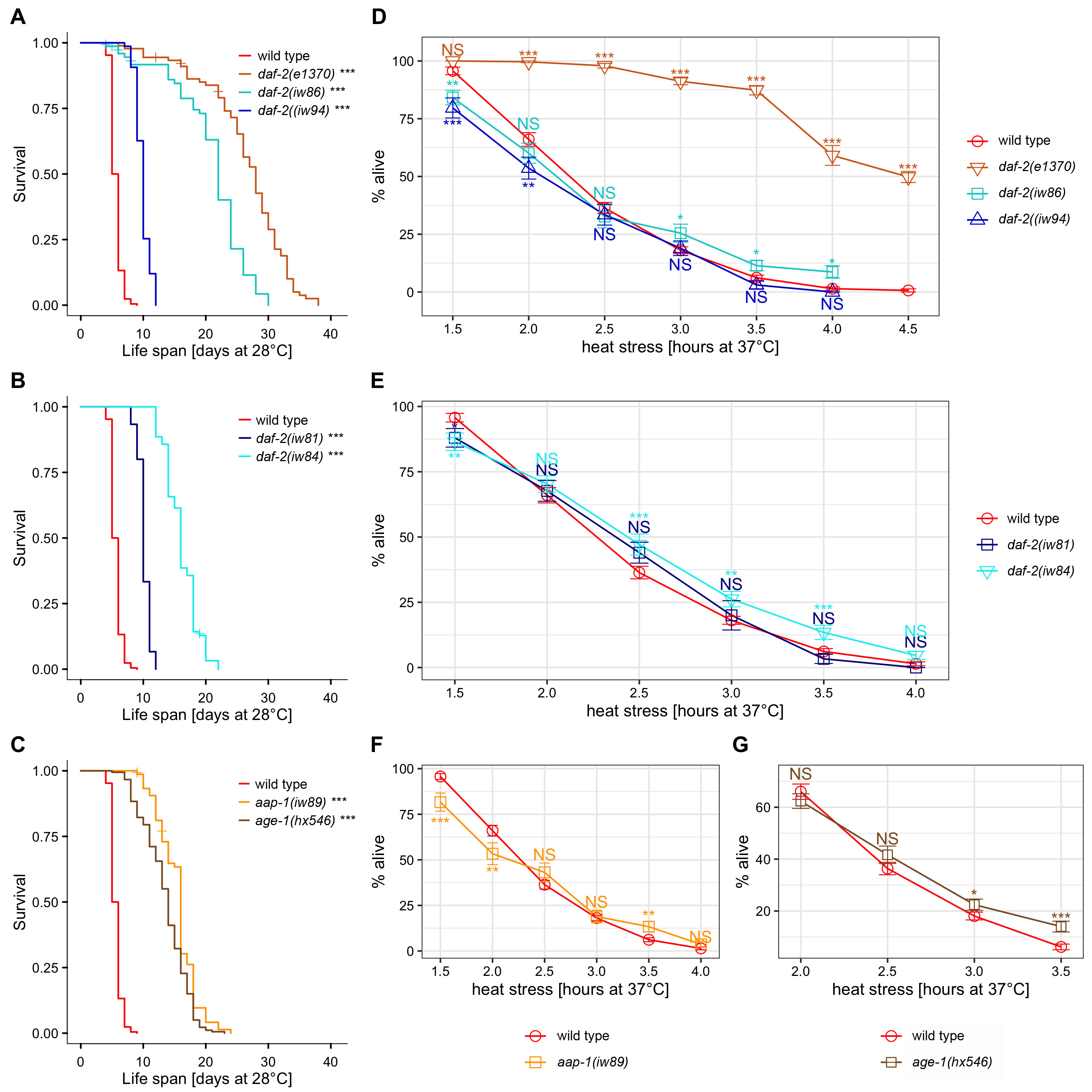

Supplement: Supplementary file 2 [file Image_2.jpg]

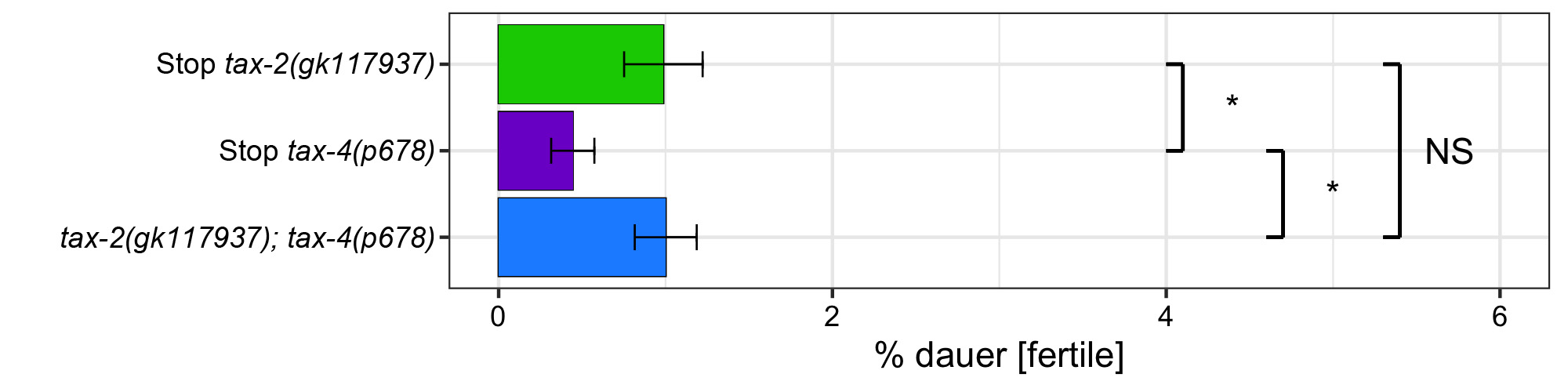

Supplement: Supplementary file 3 [file Image_3.jpg]

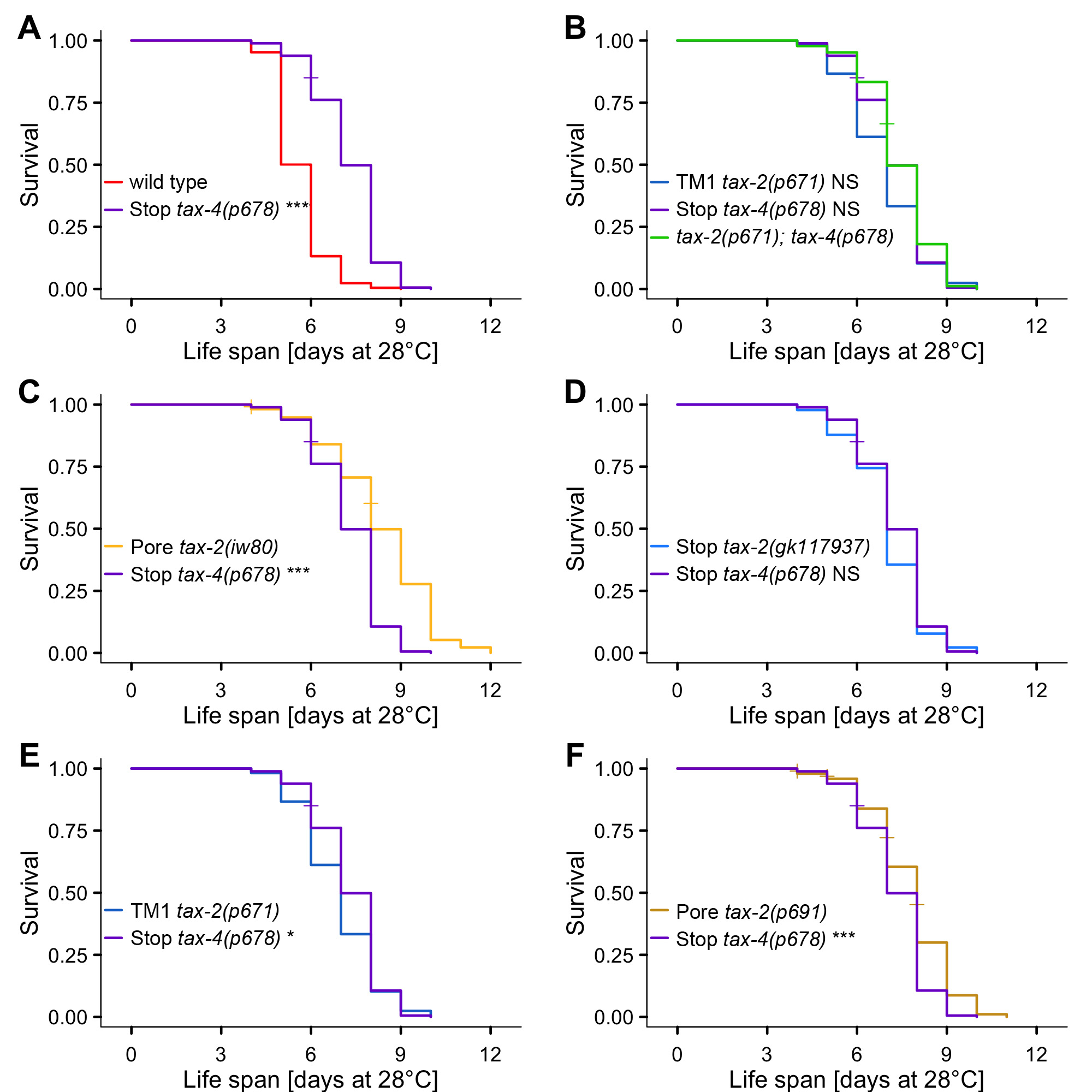

Supplement: Supplementary file 4 [file Image_4.jpg]

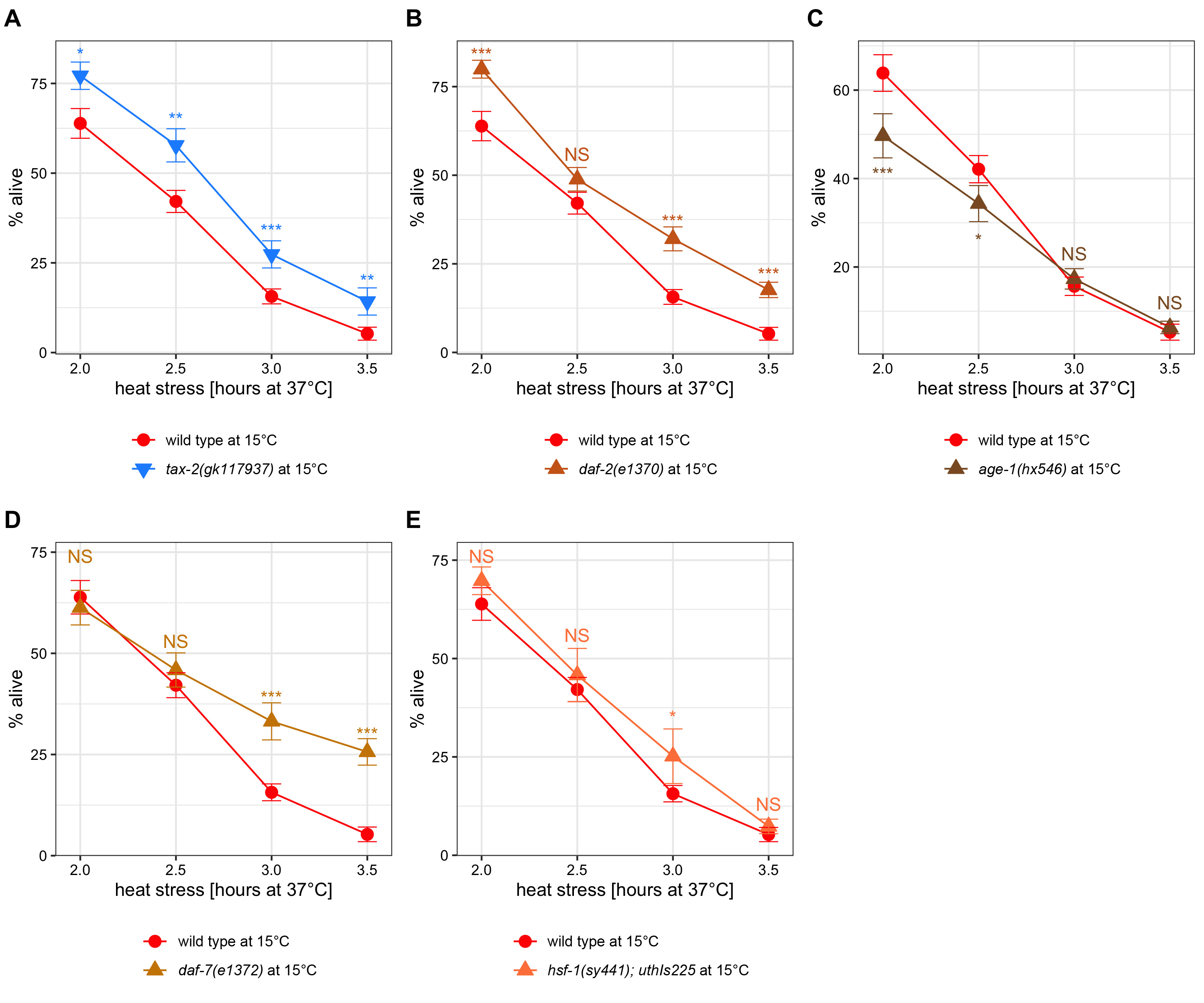

Supplement: Supplementary file 5 [file Image_5.jpg]

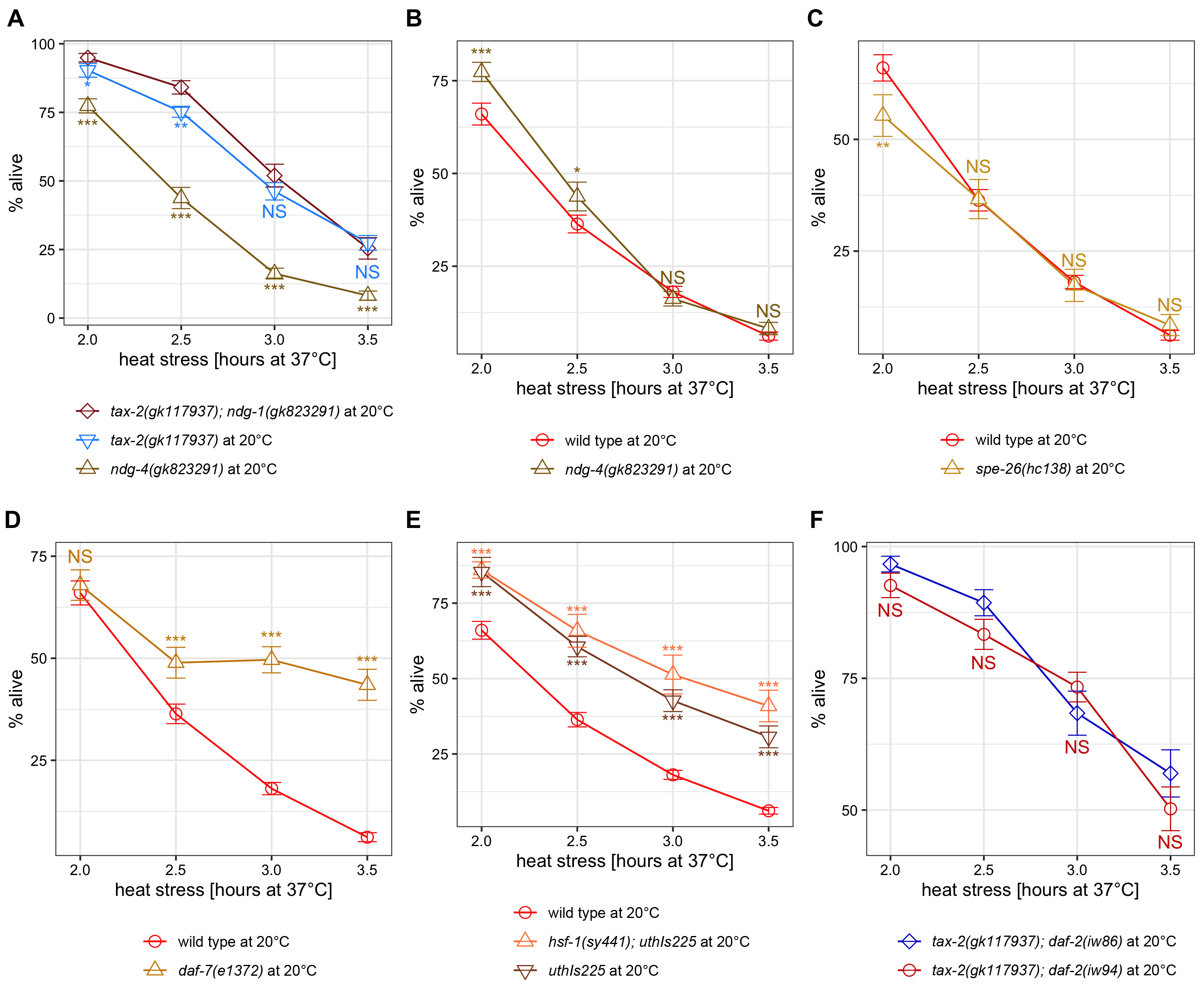

Supplement: Supplementary file 6 [file Image_6.jpg]

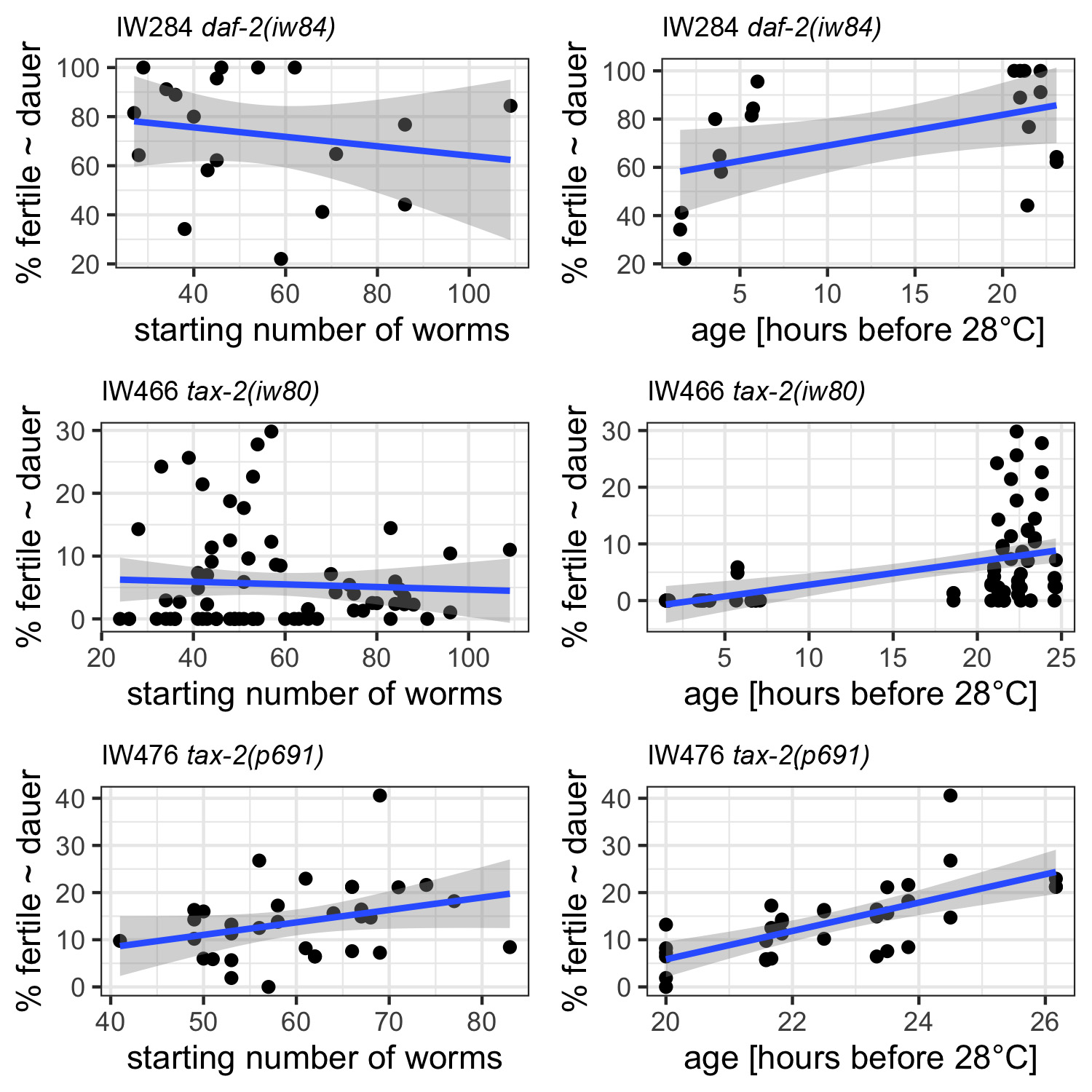

Supplement: Supplementary file 7 [file Image_7.jpg]

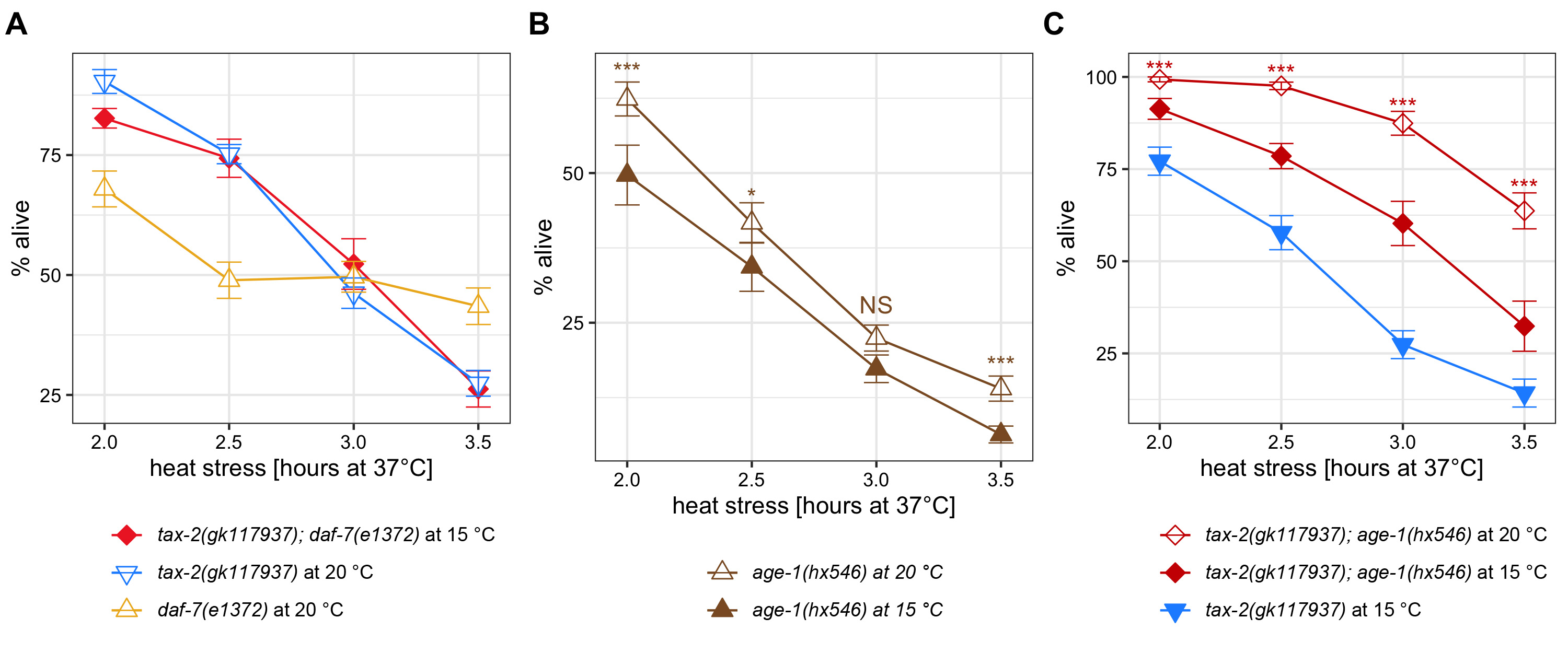

Supplement: Supplementary file 8 [file Image_8.jpg]

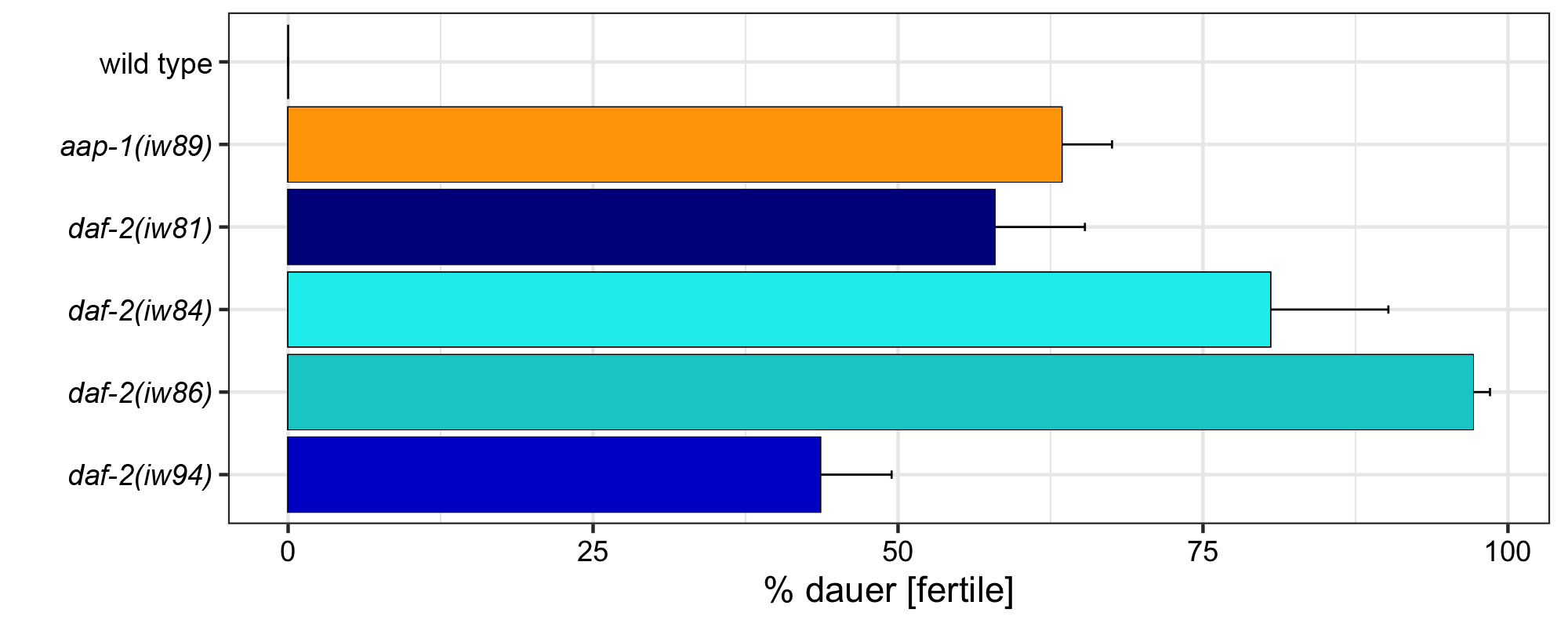

Supplement: Supplementary file 9 [file Image_9.jpg]
